# Environmental regulation, R&D investment, and green technology innovation in China: Based on the PVAR model

**DOI:** 10.1371/journal.pone.0275498

**Published:** 2022-10-03

**Authors:** Yueting Zhang, Huaichao Chen, Zhimin He

**Affiliations:** School of Economics and Management, Taiyuan University of Technology, Taiyuan, Shanxi, China; Szechenyi Istvan University: Szechenyi Istvan Egyetem, HUNGARY

## Abstract

The unreasonable economic development model of human beings has caused the environmental pollution problem to become increasingly serious. In order to achieve a positive relationship and interaction between environmental regulation, research and development (R&D) investment, and green technology innovation, and effectively solve the “strange circle” problem between high-quality economic development and environmental pollution in China and even the world, this paper takes the panel data of industrial enterprises above designated size in Chinese mainland 31 provinces from 2009 to 2019 as a research sample. The comprehensive index of R&D investment and green technology innovation was established by the entropy method, and the panel vector autoregressive (PVAR) model was constructed from the dynamic endogenous perspective, and the dynamic interaction and regional heterogeneity between environmental regulation, R&D investment, and green technology innovation were empirically analyzed by using impulse response function and variance decomposition. We obtain the following findings: (1) Environmental regulation has a two-way interaction relationship with R&D investment and green technology innovation, and R&D investment has a promotion effect on the “green degree” of technological innovation, but its role is still weak and has lagging characteristics. (2) There is significant regional heterogeneity in the dynamic responses of the eastern, central and western parts of China. (3) In the long run, environmental regulation has a “negative crowding out effect” on R&D investment in the central region, and the phenomenon of “central collapse” still exists but will gradually weaken. Environmental regulation has a “positive innovation compensation effect” on green technology innovation. Green technology innovation and R&D investment have an obvious “Pareto improvement” effect on environmental regulation, especially in the eastern region. The conclusions of this study help to clarify the dynamic interaction between environmental regulation, R&D investment, and green technology innovation, further improve environmental regulatory policies and green technology innovation R&D decision-making, and provide an effective way to achieve green and sustainable development in China and other parts of the world.

## 1. Introduction

The irrational economic development model of human beings has led to an increasingly serious problem of environmental pollution [1]. As a country with the most complex natural environment and socio-economic conditions in the world, environmental problems have become an important factor restricting China’s sustainable development [2,3]. According to the BP World Energy Statistics Review and the Global Carbon Project report, in 2020, China is one of the world’s largest energy consumers, accounting for 28% of the global total in carbon emissions, in addition, China ranks last in the global ranking of environmental performance indicators. Compared with other countries, the contradiction between environmental protection and sustainable economic development caused by China’s environmental constraints and rigid demand for economic growth is particularly prominent and has become an urgent problem to be solved [4]. In order to solve the problem, China’s 14th Five-Year Plan proposes to promote green development, strengthen the legal and policy guarantees for green development, jointly promote pollution reduction and carbon reduction, promote the comprehensive green transformation of economic and social development, and build a beautiful China [5]. It can be seen that green technology innovation combined with the characteristics of "innovation" and "green" is an important measure to break out of the strange circle of "high-quality economic development—environmental pollution," as well as an important driving force for the green transformation of economic and social development [6, 7]. At the same time, environmental regulation and R&D investment play a crucial role in promoting green technology innovation [8–14]. In this context, it is necessary to study the relationship between environmental regulation, R&D investment, and green technology innovation, which is of great significance for coordinating the coordinated development of the economy and the environment, reducing carbon emissions, and achieving a “win-win” situation of high-quality economic development and high-level ecological environmental protection in China and even other parts of the world.

Green technology innovation refers to the research and development of a technology development system that is conducive to saving resources, preventing pollution, saving energy and reducing emissions, improving energy efficiency and low-carbon cycle, which pays attention to technological development and pollution control, and jointly promotes the protection of the ecological environment while promoting technological progress and high-quality economic development. R&D investment is the primary element to stimulate green technology innovation, and the improvement of R&D investment will accelerate the speed of technological progress [8, 9]. However, enterprises implementing green technology innovation should not only bear the economic losses caused by knowledge spillovers but also bear the economic costs caused by negative environmental externalities, which makes it difficult for enterprises to carry out green technology innovation spontaneously; thus, government intervention is the key to promoting green technology innovation [10, 11]. For example, by formulating relevant environmental policies, we can solve the environmental externalities of green technology innovation, compensate for knowledge spillovers economically, reduce the possible economic losses caused by knowledge spillovers, internalize negative environmental externalities, encourage enterprises to carry out green technology innovation, and create double benefits for the economy and the environment [12–14]. On this basis, this paper examines the relationship between environmental regulation, R&D investment, and green technology innovation in order to encourage scholars to pay attention to the problems and related knowledge of green technology innovation, achieve the “win-win” goal, and elucidate the endogenous interaction mechanism among the three.

Through reviewing the existing literature, it can be found that studies on environmental regulation, R&D investment, and green technology innovation focus mostly on the following three perspectives: The first examines the existence of the “Porter hypothesis” between environmental regulation and innovation in green technology [15–17]. The second aspect is to study whether the impact of R&D investment on green technology innovation is a suppression effect or a promotion effect [18–21]. The third objective is the relationship between environmental regulation and R&D investment on green technology innovation [22–25]. It is not difficult to observe that in existing research, scholars focus on the dual impact of environmental regulation and R&D investment on green technology innovation, while ignoring the reverse impact of green technology innovation on environmental supervision and R&D investment, that is, the lack of environmental regulation, the dynamic relationship between R&D investment and green technology innovation.

According to existing literature, we believe there is a complex dynamic interaction between environmental regulation, R&D investment, and green technology innovation. On the one hand, government environmental regulation interacts with R&D investment by enterprises and is likely to affect the level of green technology innovation to variable degrees [26]. On the other hand, green technology advances will to a certain extent, encourage policymakers to increase the intensity of environmental regulation to the extent consistent with the existing level of technology, and make various factors of enterprise R&D investment more inclined to the “green” direction, resulting in a new cycle of technological advancement and environmental protection.

Then, what is the dynamic relationship between environmental regulation, R&D investment, and green technology innovation, and whether there is a certain lag period in this dynamic relationship? In addition, China is a diversified region, and there are huge gaps in environmental complexity and R&D investment levels between different regions. Will this situation cause differences in the interaction among the three? What are the different features of the eastern, central, and western regions of China? Clearly, these are urgent problems to be discussed at present, and the purpose of this paper is to answer the above questions. Discussing the above issues is conducive to a deep understanding of the nature of green transformation, making the research results more in line with the actual situation of green technology innovation, and providing more targeted guidance for environmental governance practices in China and even other developing countries and regions of the world. Therefore, we take panel data of 31 provinces in mainland China from 2009 to 2019 as the research sample. Based on the comparative perspective of the eastern, central and western regions, this paper analyzes the dynamic interaction among environmental regulation, R&D investment and green technology innovation by using the entropy method, PVAR model, impulse response function and variance decomposition. The empirical results show that the dynamic response relationship among them has obvious regional differences.

This paper provides at least three major contributions to the literature. First, the results contribute to our understanding of the relationship between environmental regulation, R&D investment, and green technology innovation. The existing literature mainly focuses on the static unidirectional interaction relationship between two variables and lacks of research on the three variables within the same framework. Second, the PVAR model is used to investigate the interaction relationship and the dynamic impact path, while the PVAR methodology outperforms the traditional VAR technique by removing any serious bias in the estimation. Third, we divide China into eastern, central and western regions and examine the heterogeneous impacts of different regions. In this way, with a view to formulating appropriate environmental regulation policies according to the characteristics of various regions in China, promoting green technology innovation in various regions in China, and achieving a “win-win” between high-quality economic development and high-level ecological environment protection.

The structure of the other parts of the article is arranged as follows: Section 2 reviews the related literature. Section 3 explains the empirical model, econometric methodology, and data employed. Section 4 presents the empirical results and interprets the findings, while the final section concludes the discussion.

## 2. Literature review

### 2.1. Environmental regulation and green technology innovation

At present, the relationship between environmental regulation and green technology innovation is an established hot topic in academic circles. However, there has been a long-standing debate on whether environmental regulation can promote green technology innovation. The existing research has initially formed three different viewpoints. First, environmental regulation inhibits green technology innovation. Some scholars are influenced by the theory of the “restriction hypothesis” in neoclassical economics [27], according to which environmental regulation will increase the cost and economic burden of pollution control of enterprises. Under the condition of constant production technology conditions, R&D investment constitutes a “crowding out effect”, weakening technological innovation capability and inhibiting green technology innovation of enterprises. For example, Milani [28] investigated the R&D data of 21 manufacturing industries in 28 Organization for Economic Co-operation and Development (OECD) countries and found that industries with more intensive pollution would reduce R&D activities and innovation behavior when facing enhanced environmental regulations.

Second, environmental regulation promotes green technology innovation [29–31]. Influenced by the Porter hypothesis [15], scholars who hold this view posit that the “innovation compensation effect” produced by environmental regulation is more than the “cost crowding effect”. Strict and appropriate environmental regulation policies can reduce or even offset the cost of pollution control and encourage the occurrence of the environmental protection innovation behavior of enterprises. For example, Jaffe and Palmer [16] introduced the Porter hypothesis and found that environmental regulation had a significant promotion effect on enterprises to enhance environmental protection research and development. This result indicates that strict environmental protection policies can fully stimulate the technological innovation of enterprises.

Third, there is uncertainty in the relationship between environmental regulation and green technology innovation [32]. The “comprehensive view” theory holds that the relationship between them is uncertain due to different conditions. Li and Shen [18] found that environmental regulation and technological innovation had a threshold effect rather than a simple linear relationship, and the relationship between them was affected by regional differences. At the same time, Albrizio et al. [33] and Dong et al. [34] believed that the relationship between environmental regulation and green technology innovation might be heterogeneous among enterprises, industries and regions.

### 2.2. R&D investment and green technology innovation

Whether the impact of R&D investment on green technology innovation is an inhibiting or a promoting effect? Some scholars support the view that R&D investment can significantly promote technological innovation, especially for late-developing countries, and strengthening the independent R&D of enterprises will have a significant positive impact on domestic technological progress [18]. Park [20] and Romer [21] empirically tested the positive effect of R&D investment on green technology innovation from the perspective of enterprise R&D investment and government R&D investment, respectively. However, they lack dynamic research on R&D investment in the green technology innovation development process. Bai et al. [35] verified that government R&D investment was an important source of green technology innovation through the propensity score matching method, and with the deepening of industrial pollution, green technology innovation ability was more affected by government R&D investment. However, some studies have shown that an increase in R&D investment might eventually show a decrease in total factor productivity. Different from other studies, Wang et al. [36] took enterprise scale as the threshold variable, established a super-SBM threshold model, and analyzed the double threshold effect of R&D investment intensity on green innovation efficiency. The results showed that the effect of R&D investment on green technology innovation varied with the scale of the enterprise. As the scale of the enterprise expands, the effect of R&D investment on green technology innovation changes from the initial inhibition effect to the promotion effect.

### 2.3. Environmental regulation, R&D investment, and green technology innovation

At present, research on the combination of environmental regulation, R&D investment and green technology innovation is relatively scarce, and only a few studies examine the unidirectional influence and nonlinear effect of environmental regulation and R&D investment on green technology innovation [22]. On the one hand, external factors play an important role in green technology innovation ability through internal factors. This is manifested in the fact that enterprises are facing increasingly comprehensive and strict environmental regulations. Original competitiveness will be impacted, and production technology activities cannot use the current environmental requirements. Enterprises will gain new economic benefits by increasing technological innovation. That is, with the increase in environmental regulation, enterprises will increase their corresponding R&D investment and further enhance their green technology innovation ability [24, 25]. On the other hand, under different R&D investment levels, the impact of environmental regulation on green technology innovation varies, which shows that under medium and low intensity R&D investment, environmental regulation has a significant inhibitory effect on green technology innovation. With the increase in R&D investment intensity, environmental regulation has a significant positive impact on green technology innovation. Thus, the inconsistent findings in the literature remain an important issue to investigate.

In summary, we find that although there are some studies in the existing literature on environmental regulation, R&D investment and green technology innovation and that a large number of academic achievements have been accumulated, there continue to be the following research gaps. (1) environmental regulation, R&D investment and green technology innovation are rarely included in the same framework, and only the one-way relationship between variables is analyzed. (2) Ignoring the endogeneity and time lag of model estimation will easily lead to nonobjective conclusions. (3) Without dynamic and static double-angle tests, it is difficult to analyze the deep-seated dynamic interaction, explain the dynamic relationship between variables strictly and effectively, and further analyze the differences in this dynamic relationship in different regions of China. To compensate for the lack of existing research, we measured the level of environmental regulation, R&D investment, and green technology innovation in 31 provinces in the Chinese mainland. Based on comparative perspectives of eastern, central and western regions, a PVAR model is established to verify the effect of the initial investment of variables on themselves and other variables, determine its lag period, and analyze the long-term and short-term dynamic interaction effects between the three through the pulse effect graph and variance decomposition results to promote harmonious interaction between the three. Our findings are applicable to China and other developing countries or regions around the world, and can be used to improve environmental regulatory policies and green technology innovation R&D decisions, promote the coordinated development of the economy and the environment, and reduce carbon emissions. Further, to achieve a “win-win” economic development and ecological environmental protection, as well as green and sustainable development to provide an effective way.

## 3. Methodology and data

### 3.1. Entropy method

Compared to the subjective assignment method, the entropy method, as an objective assignment method, can effectively avoid the influence of human factors, and has been applied to the assignment calculation in various fields and has been recognized by the academic community. In order to make the research results more scientific and reliable, this paper uses the entropy method to construct a comprehensive index of R&D investment and green technology innovation. According to the information entropy principle and the research method, the time variable is added to the entropy method, and the index weights of enterprises in each province are calculated in turn. Then, the comprehensive evaluation scores of multiple indices are formed as a whole. The implementation process is illustrated as follows.

First, the index is nondimensionalized to reduce the influence of extreme values on comprehensive evaluation and ensure the nonnegativity of data. Data translation is carried out for positive indicators and negative indicators.

$$X_{ij}^{*}=\frac{X_{ij}-\min(X_{1j},X_{2j},\cdots ,X_{nj})}{\max(X_{1j},X_{2j},\cdots ,X_{nj})-\min(X_{1j},X_{2j},\cdots ,X_{nj})},i=1,2,\cdots ,n;j=1,2,\cdots ,m$$ (1)

$$X_{ij}^{*}=\frac{X_{ij}-\max(X_{1j},X_{2j},\cdots ,X_{nj})}{\max(X_{1j},X_{2j},\cdots ,X_{nj})-\min(X_{1j},X_{2j},\cdots ,X_{nj})},i=1,2,\cdots ,n;j=1,2,\cdots ,m$$ (2)

where *X*_ij_* represents the standardized assignment, *i* is the province, and *j* is the index. This paper represents the R&D investment index and green technology innovation index, and *X*_*ij*_ represents the original value of the index.

Second, the specific gravity *P*_*ij*_ of each index *X*_ij_^*^, the entropy value *e*_*j*_ and the difference coefficient *g*_*j*_ of the annual *j-th* index are calculated. Furthermore, the weight *W*_*j*_ of each index is obtained,

$$P_{ij}=\frac{X_{ij}^{*}}{\sum_{i}^{n}X_{ij}^{*}}(j=1,2,\cdots ,m),e_{j}=\frac{1}{\ln\;m}\sum_{i=1}^{n}P_{ij}\ln\;P_{ij},g_{j}=1-e_{j},W_{j}=\frac{g_{j}}{\sum_{j=1}^{m}g_{j}}$$ (3)

Finally, according to the standardization result *P*_*ij*_ and weight *W*_*j*_ of each index, the R&D investment comprehensive index and green technology innovation comprehensive index *S*_*it*_ of the *i*th province in the *t*th year are calculated,

$$S_{it}=\sum_{j=1}^{m}W_{j}*P_{ij}(i=1,2,\cdots ,n)$$ (4)

### 3.2. Panel vector autoregressive model

The panel vector autoregressive (PVAR) model was proposed in 1988 [16]. It is a multivariate system equation that solves the problems of endogenous variables and lag terms [37]. The time and fixed effects can be considered in the PVAR model. Using generalized moment estimation (GMM), impulse response function (IRF) and variance decomposition (FEVD) tools, it effectively describes the short-term response and long-term motion trend among variables and truly reflects the interaction effect among variables [38]. Compared with the VAR model, the PVAR model further considers the time effect and fixed effect, and the results are more accurate [39, 40]. Scholars have improved and demonstrated the estimation method of the PVAR model [38, 41, 42], and the PVAR model has matured.

Considering that the logical relationship between environmental regulation, R&D investment and green technology innovation is complex, there may be an intrinsic causal relationship between variables. In order to accurately study the dynamic correlations and regional heterogeneity between them, this paper constructs a PVAR model by incorporating environmental regulation, R&D investment, and green technology innovation into the same framework. The PVAR model is shown as Eq (5),

$$y_{it}=\alpha_{0}+\sum_{j=1}^{p}A_{j}y_{t-j}+f_{i}+d_{i}+\varepsilon_{it}$$ (5)

Expand *y*_*it*_ to obtain Eq (6):

$$y_{it}=\{\begin{array}{c} \ln\;{\mathrm{ER}}_{it}\\ \ln\;{R\&D}_{it}\\ \ln\;{\mathrm{GTI}}_{it} \end{array}$$ (6)

In Eq (5) and Eq (6), *y*_*it*_ represents the vector composed of endogenous variables of the *i-th* province in the *t-th* year, which are environmental regulation, R&D investment and green technology innovation in science and technology. The subscript *i* = (1, 2, …, 31) represents 31 provinces, *t* ranges from 2009 to 2019, *j* represents the lag order of each variable, *y*_*t-j*_ represents endogenous variables, *α*_*0*_ represents the intercept term, *A*_*j*_ represents the regression coefficient matrix, *f*_*i*_ and *d*_*i*_ represent fixed effect and time effect, and *ε*_*it*_ represents the random disturbance term. We follow the PVAR program from Love and Zicchino [38] with the calculation software *Stata16*.*0*.

### 3.3. Variable description and data sources

The statistics yearbook does not account for the total pollutant output and the industrial output value of industrial enterprises produced after 2019, based on the comprehensive consideration of the scientific rationality of data and the rigor of analysis results, this paper takes the panel data of 31 provinces in mainland China from 2009 to 2019 as the research samples. The sales revenue of new products, the full-time equivalent of R&D personnel and the internal R&D expenditures in the original data are all from the *China Science and Technology Statistical Yearbook* and *China Statistical Yearbook*. Due to the delay in updating the data in recent years, the data related to industrial pollution from 2009 to 2015 came from the *China Environmental Statistics Yearbook*. The data related to industrial pollution from 2016 to 2019 mainly came from the *China Urban Statistics Yearbook* and statistical yearbooks of various provinces. Among them, the data of very few provinces are missing, and the average trend method is used to complete them. The rest of the data come from the National Bureau of Statistics. The Specific raw data is in S1 File.

(1) Environmental regulation (ER)

Total investment in industrial pollution control scientifically reflects the results of environmental control. Most scholars use the total investment in pollution control to characterize environmental regulation. In the regional heterogeneity analysis of this paper, to effectively avoid the influence of regional output value differences on variables, and drawing on the measuring index from Zhang et al. [32], the environmental regulation index is characterized by the ratio of total investment in industrial pollution control and total regional industrial output value (regional GDP). The higher the value, the higher the pollution control cost and the greater the intensity of environmental regulation.

(2) R&D investment (R&D)

The degree of investment in R&D directly affects the innovation vitality and level of the enterprises themselves. Furthermore, it reflects the importance attached to technological innovation in this region [43]. The internal R&D expenditure and the personnel full-time equivalent of R&D comprehensively and systematically reflect the investment system of R&D. Following the Qin et al. [44] handling method, this study standardizes the personnel full-time equivalent and the internal expenditure of R&D funds of industrial enterprises above a designated size and combines them into a comprehensive index by the entropy method as the proxy variable of R&D investment. At the same time, due to the change in statistical caliber, the samples in 2010 and before are the data of large and medium-sized industrial enterprises. The samples in 2011 and after are the data of industrial enterprises above a designated size, which are basically consistent and will not cause errors in the analysis results.

(3) Green technology innovation (GTI)

Consistent with the practice of R&D investment, this study uses the entropy method to construct the comprehensive index of green technology innovation. Green technology innovation includes two parts: green process innovation and green product innovation. Green process innovation mainly reflects the reduction of total pollutant output by means of process transformation and equipment renewal and measures the green process innovation index of enterprises by the industrial added value of total pollutant output per unit. Green product innovation is mainly reflected in two aspects: economic development and environmental protection. Through independent research, design, development and manufacture of products that meet environmental requirements, the pollution problem can be effectively reduced, and the ecological environment can be protected. The ratio of new product sales revenue and total pollutant production of enterprises are mainly used to measure the green product innovation index. The specific model index system is shown in Table 1.

**Table 1:** Explanation of environmental regulation, R&D investment and green technology innovation index system.

Indicator layer	Indicator description		Specific indicators
Environmental regulation	Comprehensive strength		Total investment in industrial pollution control/total industrial output value
R&D investment	Composite index		FTE of R&D personnel
			Internal R&D expenditure
Green technology innovation	Composite index	Green process innovation	Industrial added value/total pollutant output of industrial enterprises
		Green product innovation	Sales revenue of new products of industrial enterprises/total pollutant output of industrial enterprises

## 4. Empirical results and discussion

### 4.1. Panel data stationarity test

Before building the PVAR model, to ensure the validity of the estimation results and avoid the possible “pseudoregression” phenomenon caused by nonstationary data, this paper uses the Levin-Lin-Chu (LLC) [45] and ADF-Fisher [46] methods to compare and test the data of the eastern, central and western regions. Table 2 shows that the test values of the variables in each region reject the original hypothesis of a unit root of at least 5%, and the panel data in the samples are stationary.

**Table 2:** Test results of panel smoothness.

Sample	Indicators	ER	R&D	GTI
Eastern region	LLC	-14.434***	-2.545***	-3.879***
	ADF-Fisher	45.506***	41.027***	38.821***
Central region	LLC	-3.498***	-24.315***	-3.667***
	ADF-Fisher	149.567***	84.361***	39.044***
Western region	LLC	-7.819***	-3.535***	-6.467***
	ADF-Fisher	133.383***	150.339***	41.052**
Conclusion	Whether it is stable or not	Stable	Stable	Stable

Notes: The 1%, 5% and 10% levels of significance are indicated by ***, ** and * respectively.

### 4.2. Modeling

After preliminarily clarifying that the variables in each region are a stationary sequence, the panel cointegration test is used to study whether there is a long-term equilibrium relationship between variables in various regions. Because there are at least two statistics that reject the original hypothesis that there is no cointegration relationship, the results of the cointegration test show that there is a long-term equilibrium relationship among environmental regulation, R&D investment and green technology innovation. Due to space limitations, the results of the cointegration test are no longer listed here.

The selection of lag order in the PVAR model will have a great impact on the estimation results of each statistic. The optimal lag order of each variable is determined according to the Akaike information criterion (AIC), Bayesian information criterion (BIC) and HQIC criteria (as shown in Table 3). When the optimal lag order has been chosen as 1 for the eastern, central and western regions, the PVAR model has good estimation results. Therefore, the PVAR model with lag order 1 is established according to Eq (5),

$$y_{it}=B_{0}+B_{1}y_{it-1}+f_{i}+d_{i}+\varepsilon_{it}$$ (7)

**Table 3:** Selection of lag order of PVAR model.

Sample	Hysteresis period	AIC	BIC	HQIC
Eastern region	1	-21.829*	-80.951*	-45.191*
	2	-20.539	-59.954	-36.114
	3	-15.827	-35.534	-23.614
Central region	1	-34.937*	-85.461*	-54.030*
	2	-16.183	-49.865	-28.911
	3	-5.068	-21.909	-11.432
Western region	1	-31.317*	-92.787*	-55.788*
	2	-17.978	-58.958	-34.293
	3	-6.799	-27.289	-14.956

Notes: The upper corner mark * indicates the best lag period

### 4.3. Granger causality test

Theoretically speaking, R&D investment is the cause of green technology innovation, and the incentive effect produced by green technology innovation also promotes R&D investment. Therefore, green technology innovation may also be the cause of R&D investment. Does the actual data support this? Does environmental regulation promote green technology innovation? Does green technology innovation inhibit the increase in total investment in industrial pollution control? The Granger causality test must be finished to answer the above three questions. Previous research shows that panel data are in a stationary state. Therefore, the Granger causality test can be carried out.

Table 4 shows that there is a two-way Granger causality relationship between environmental regulation and green technology innovation in the three regions. This indicates that there is a significant two-way interaction between them. Except for the eastern region, there is two-way Granger causality between environmental regulation and R&D investment. R&D investment in the eastern region is not the Granger cause of environmental regulation, which indicates that R&D investment has a weak impact on environmental regulation in this region. There is only one-way Granger causality between green technology innovation and R&D investment, which illustrates that the promotion of the green technology innovation level can promote the further investment of research and development funds. R&D investment has little influence on the greenness of technological innovation.

**Table 4:** Granger causality test results.

Sample	Be explained Variable	Explanatory variable	*Chi2*	Sample	Be explained Variable	Explanatory variable	*Chi2*	Sample	Be explained Variable	Explanatory variable	*Chi2*
Eastern region	H_GTI	h_ER	19.479***	Central region	h_GTI	h_ER	143.856***	Western region	h_GTI	h_ER	8.123***
		h_R&D	0.641			h_R&D	28.779			h_R&D	2.582
		ALL	21.477***			ALL	147.211***			ALL	12.201***
	H_ER	h_GTI	1.524*		h_ER	h_GTI	32.894***		h_ER	h_GTI	9.893***
		h_R&D	1.958*			h_R&D	20.283***			h_R&D	7.450***
		ALL	10.585***			ALL	34.570***			ALL	10.612***
	H_R&D	h_GTI	0.288*		h_R&D	h_GTI	30.233***		h_R&D	h_GTI	6.261**
		h_ER	24.309***			h_ER	47.131***			h_ER	32.774***
		ALL	28.102***			ALL	47.215***			ALL	44.941***

Notes: The upper corner marks ***, ** and * indicate that the statistical test is significant at the levels of 1%, 5% and 10%, respectively.

### 4.4. PVAR model analysis

#### 4.4.1. GMM estimation

The Helmert process is used to process the data and eliminate the individual fixation effect by the first-order forward difference method. Elimination of the time fixed effect by the mean difference method will weaken the fixed effect of the sample and avoid the causing of coefficient estimation error during the PVAR estimation process. With the optimal lag order of 1, the generalized method of moments (GMM) is used for parameter estimation to improve the effectiveness of coefficient estimation. The model estimation results are shown in Table 5.

**Table 5:** Estimation results of GMM parameters of PVAR model.

Module	Module 1			Module 2			Module 3		
Variable	h_GTI			h_ER			h_R&D		
Sample	Eastern	Central	Western	Eastern	Central	Western	Eastern	Central	Western
	Coefficient/P	Coefficient/P	Coefficient/P	Coefficient/P	Coefficient/P	Coefficient/P	Coefficient/P	Coefficient/P	Coefficient/P
h_GTI L1.	0.550*** (0.082)	0.303*** (0.042)	0.540*** (0.091)	-1.925 (1.559)	-0.291*** (0.051)	-1.533** (0.487)	0.018 (0.033)	-0.301*** (0.055)	-0.287* (0.115)
h_ER L1.	0.013*** (0.003)	0.506*** (0.042)	0.061* (0.022)	0.237*** (0.070)	0.406*** (0.078)	0.181* (0.087)	0.098*** (0.020)	-0.420*** (0.061)	0.128*** (0.022)
h_R&D L1.	0.050 (0.062)	-0.109 (0.086)	0.171*** (0.032)	-1.706 (1.219)	-0.252*** (0.056)	-0.700** (0.256)	0.744*** (0.034)	0.531*** (0.047)	1.026*** (0.076)

Notes: (1) “h_” denotes the result of eliminating the fixed effect by the Helmert transformation. (2) “L1.” means the lag of the first order. (3) The 1%, 5% and 10% levels of significance are indicated by ***, ** and *, respectively. (4) The standard error values are indicated in brackets.

In Module 1 of Table 5, green technology innovation is taken as an explanatory variable. It is found that environmental regulation significantly promotes green technology innovation in lag order 1, with strong timeliness. According to the classification of samples by region, it can be seen that the promotion of environmental regulation on green technology innovation in the central region is higher than in other regions. There are relatively more carbon emissions and pollutants in the central region, which stimulates regional green technology innovation. The R&D investment of lag order 1 in the western region significantly promotes green technology innovation, while the influence of other regions has not yet reached a significant degree. This indicates an evident catch-up effect in the western region.

In Module 2 of Table 5, environmental regulation is taken as the explained variable. This indicates that green technology innovation and R&D investment of lag order 1 both inhibit environmental regulation. The direct inhibitory effect of green technology innovation on environmental regulation is higher than that of R&D investment. This shows that the increase in green technology innovation and R&D investment in each region reduces the cost of pollution control and promotes environmental improvement. The benefit of green technology innovation promotion on environmental improvement is better than that of the increase in research and development investment on environmental improvement.

In Module 3 of Table 5, R&D investment is taken as the explained variable. It is found that green technology innovation and environmental regulation have a significant impact on R&D investment. However, the green technology innovation of lag order 1 in the central and western regions has a restraining effect on R&D investment. This inhibition effect is more significant in the central region. At the same time, the environmental regulation of lag order 1 in the central region also has a significant inhibition on R&D investment. Compared with other regions, the unreasonable methods of environmental regulation and the lack of a “green degree” of technological innovation in the central region weaken its positive impact on R&D investment.

#### 4.4.2. Impulse response functions

Because there are many regression coefficients in the PVAR model, it is difficult to reflect the dynamic conduction path and interaction effect among variables in different regions in the future. Therefore, based on 1,000 Monte-Carlo simulations, the interaction relationship among the eastern, central and western regions in the next six periods in the 95% confidence interval is described by the impulse response function. Impulse response diagrams measure the short-term, purely unilateral impact of one variable on another subject to a unit standard deviation shock. It can be seen from the figure that the response of each variable to the impact from itself is a significant positive effect. However, with the passage of time, this positive effect gradually decreases, and the promotion degree gradually weakens.

(1) Environmental regulation plays a positive role in promoting green technology innovation, and the “positive innovation compensation effect” is evident. Green technology innovation has a negative impact on environmental regulation, especially in central China.

**Fig 1:**
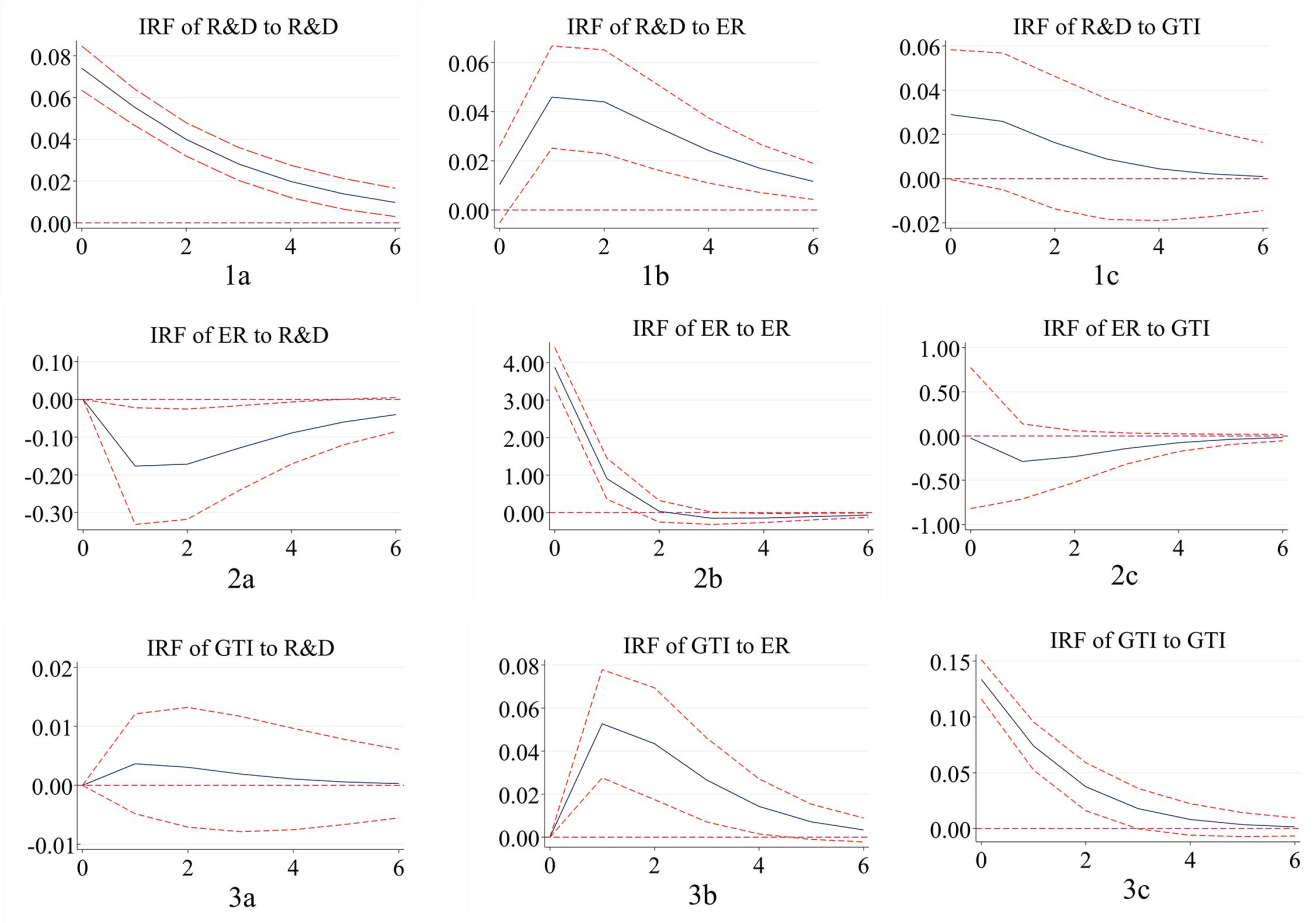
Impulse response in eastern China. Notes: The horizontal axis represents the number of lag periods (years), the middle curve is the impulse response function curve, and the upper and lower curves represent the 95% confidence interval.

**Fig 2:**
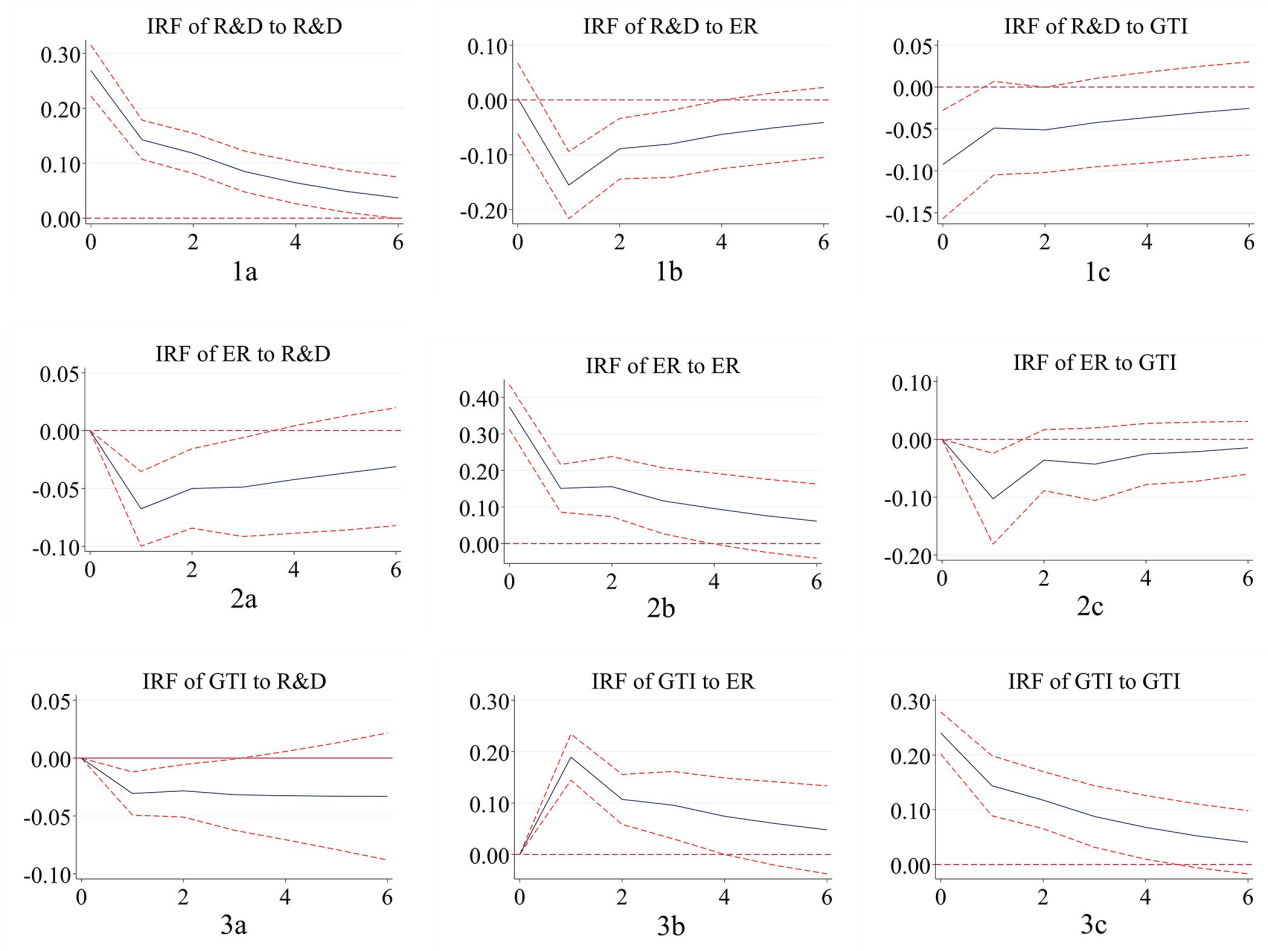
Impulse response in central China. Notes: The horizontal axis represents the number of lag periods (years), the middle curve is the impulse response function curve, and the upper and lower curves represent the 95% confidence interval.

**Fig 3:**
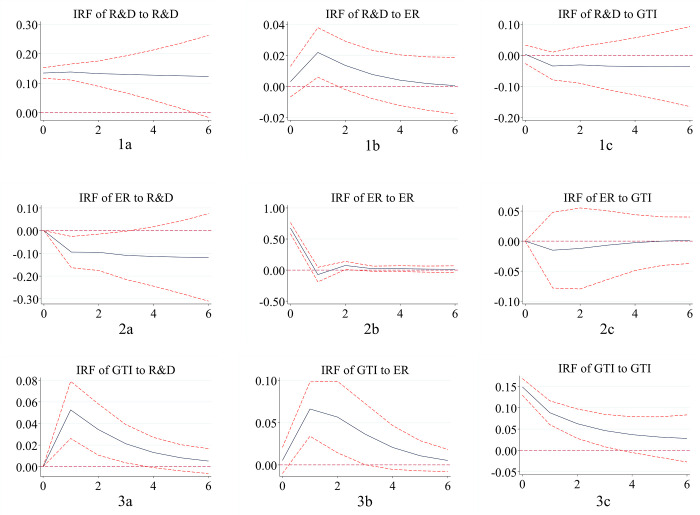
Impulse response in western China. Notes: The horizontal axis represents the number of lag periods (years), the middle curve is the impulse response function curve, and the upper and lower curves represent the 95% confidence interval.

Comparing Figs 3B and 2C in Figs 1–3, it is found that environmental regulation plays a positive role in promoting green technology innovation. Green technology innovation inhibits the increase in environmental regulation intensity. As an error disturbance term, the impact fluctuation path of environmental regulation on green technology innovation in the eastern, central and western regions is changed according to an inverted “V” trend. All of them reach their peak in the first period in the future. Then, it shows a slow decline approaching zero, indicating that the innovation compensation effect of environmental regulation on green technology innovation is greater than the compliance cost effect. This has a positive role in promoting green technology innovation ability. This conclusion is consistent with most research results and supports the Porter hypothesis. In the central region, it is more remarkable that green technology innovation has a negative impact on environmental regulation. It reaches the strongest impact value of negative 0.1 in future phase 1. With the progress of green technology, enterprises are willing to adopt more advanced technologies to meet environmental needs, and the cost of industrial pollution control will decrease. The intensity of environmental protection will be strengthened. Furthermore, compared with other regions, the central region will produce more environmental improvement effects under the premise of the same green technology innovation.

(2) R&D investment has a positive effect on green technology innovation in the eastern and western regions, with an evident catch-up effect in the western region. Green technology innovation has a negative impact on R&D investment in the central and western regions, where the phenomenon of “central collapse” is apparent.

Comparing Figs 3A and 1C in Figs 1–3, respectively, R&D investment in the eastern and western regions fluctuates positively on green technology innovation. The performance of the western region is more remarkable and rapid, reaching a peak of 0.05 in the next period 1. The positive promotion of the eastern region reaches the strongest value in the next period 1. However, the strongest value is only close to 0.005, which is strong in the early stage of fluctuation and tends to be stable in the later stage. At the same time, the central region has a negative impact, and R&D investment has not formed a promotion effect on green technology innovation. Due to the continuous implementation of the western development strategy, the catch-up effect in the western region is evident. In the eastern region, basic innovation is the main factor, while technical barriers and technical blockades are difficult challenges. Green technology innovation has a time lag. The opening thought in the central region is relatively lagging behind, and the attention to green technology innovation and the R&D investment of enterprises is insufficient. As a result, it is difficult to improve the level of green technology innovation in a short period of time. Green technology innovation in eastern China has a positive impact on R&D investment, and there is a downward trend. Green technology innovation plays a negative role in R&D investment in the central and western regions. The intensity is higher in the central region than in the western region. This indicates that there is still a phenomenon of “central collapse”. As a policy depression region, the central region is facing the challenges of the prosperity of the east and the development trend of the west. The momentum and level of innovation and development are lower than those in the east and west. The investment in scientific research is constantly inhibited. However, in the long run, the inhibitory effect of green technology innovation on R&D in the central region will continue to weaken.

(3) Environmental regulation has a positive effect on R&D investment in the eastern and western regions, while there is a “negative crowding out effect” in the central region. R&D investment has a restraining effect on environmental regulation, and the “Pareto improvement” is evident.

Comparing Figs 2A and 1B in Figs 1–3, environmental regulation in the eastern and western regions has a positive role in promoting R&D investment. This result indicates that an increase in environmental regulation intensity is helpful for encouraging enterprises to increase R&D capital investment. Most of the eastern regions focus on basic research, which is difficult to develop. More money needs to be invested in ensuring a “win-win” outcome between the environment and the economy. The western region shows a certain late catch-up in R&D investment due to the increasing emphasis on the environment in western China. In addition to the increased severity of the central government’s regional environmental regulations, the central region shows a trend of first decreasing and then increasing. Environmental regulation can stimulate the R&D investment of enterprises. However, due to the severe pollution problem in central China, the existence of environmental regulation has a “negative crowding out effect” on R&D investment in the short term. In the long term, this negative effect will be significantly weakened. In the eastern region, the inhibition intensity of R&D investment on environmental regulation is higher than that in the western region, and it is the lowest in the central region. All of them reach the lowest value in the first period in the future. The increase in R&D investment will promote the decrease in pollution control costs, and the more R&D investment there is, the better the environment will develop, and “Pareto improvements” will take place.

#### 4.4.3. Variance decomposition

Variance decomposition measures the relative importance of variables by analyzing the contribution of different endogenous variables to structural influence. To evaluate the long-term interaction among environmental regulation, R&D investment and green technology innovation more accurately, based on PVAR estimation and impulse response analysis, variance decomposition is further carried out on the panel data. On the whole, Table 6 shows that environmental regulation, R&D investment and green technology innovation have the greatest contribution rate to their own impact. There is a significant two-way interaction effect between environmental regulation and green technology innovation. The contribution rate of R&D investment to green technology innovation is relatively weak, and the overall green innovation efficiency in central China is low.

**Table 6:** Results of variance decomposition.

Be explained Variable	Number of periods	Eastern region			Central region			Western region		
		GTI	ER	R&D	GTI	ER	R&D	GTI	ER	R&D
GTI	10	0.815	0.183	0.002	0.599	0.384	0.017	0.758	0.178	0.064
ER	10	0.011	0.986	0.003	0.282	0.673	0.045	0.127	0.705	0.168
R&D	10	0.025	0.353	0.622	0.101	0.257	0.642	0.062	0.031	0.907
GTI	20	0.815	0.183	0.002	0.597	0.385	0.018	0.621	0.327	0.052
ER	20	0.010	0.986	0.004	0.282	0.672	0.046	0.121	0.591	0.288
R&D	20	0.025	0.353	0.622	0.101	0.258	0.641	0.071	0.021	0.908

From the perspective of the relationship between green technology innovation, environmental regulation and R&D investment, the contribution rate of environmental regulation to green technology innovation is generally high in all regions of China. In the 10th period, there was a stable contribution rate of 18.3% in the eastern region. The contribution rate of green technology innovation in central China was stable at 38.5% in the 20th period. In western China, it showed a significant soaring trend and finally stabilized at 32.7% in the 20th period. Similarly, in central China, the contribution rate of green technology innovation to environmental regulation was higher than that in other regions, reaching 28.2% in the 10th period. The lowest rate is in eastern China, which gradually decreases its intensity with time, reaching a stable 1% in the 20th period. Compared with other regions, the contribution rate of green technology innovation to R&D investment in the central region is the highest, reaching a stable 10.1% in the 10th period, while the contribution rate of green technology innovation to R&D investment in the eastern region and western region is 2.5% and 7.1%, respectively, in the 20th period. Similarly, the contribution rate of R&D investment to green technology innovation has obvious regional differences, showing the contribution rate of green technology innovation. The values for the western region, central region and eastern region are 5.2%, 1.8% and 0.2%, respectively.

From the perspective of the relationship between environmental regulation and R&D investment, the contribution rate of environmental regulation to R&D investment is the highest in the eastern region, reaching a steady 35.3% in the 10th period. It is expected to continue to rise with the increase in the number of periods, and the contribution rate in the central region is greater than that in the western region. The contribution rate of R&D investment to environmental regulation is generally low in all regions of China, which is consistent with the results of the impulse response function. Among them, the contribution rate of the western region is the highest, reaching a stable 28.8% in the 20th period, which is consistent with that of the central region. The contribution rate of the eastern region is increasing, reaching a stable state in the 20th period, with contribution values of 4.6% and 0.4%, respectively.

## 5. Conclusion and policy implications

In order to solve the contradiction between high-quality economic development and environmental protection, we must accurately comprehend the relationship between environmental regulation, R&D investment, and green technology innovation. Currently, the majority of empirical research on environmental regulation, R&D investment, and green technology innovation focuses on testing the static one-way relationship between the two variables, while ignoring the reverse effect, and lacks research on the two-way dynamic relationship between the three variables in the same framework. To fill this gap, this paper takes the panel data of 31 provinces in mainland China from 2009 to 2019 as the research sample. Based on the comparative perspective of the eastern, central and western regions, this paper analyzes the dynamic interaction among environmental regulation, R&D investment and green technology innovation by using the entropy method, PVAR model, impulse response function and variance decomposition. The empirical results show that the dynamic response relationship among them has obvious regional differences. The specific conclusions are as follows.

First, we found that environmental regulation and green technology innovation have a significant two-way interaction effect. Environmental regulation promotes green technology innovation, and the eastern, central and western regions will reach the maximum in the first phase in the future; this “positive innovation compensation effect” is more significant in the central region. At the same time, we also found that green technology innovation has a restraining effect on environmental regulation. Similarly, the contribution rate of the restraining effect of green technology innovation on environmental regulation in the central region is higher than that in other regions. The performance in the eastern region is the least evident. As the resource base of China, compared with other regions, environmental regulation in the central region can guide the factors of technological progress in a green direction. It encourages regional industrial transformation and economic structure upgrading, and produces greater environmental improvement effects on the premise of the same green technological innovation. The research results support the findings of Zhang and Chen [40] and Wu et al. [39] to a certain extent, and compensate for the shortcomings of Nakhli et al. [47] by incorporating environmental regulations into the research framework.

Second, except for the weak influence of R&D investment on environmental regulation in the eastern region, there is a significant two-way interaction between environmental regulation and R&D investment. the research results support the findings of Wu et al. [39] to a certain extent. It is found that environmental regulation has different positive effects on R&D investment in the eastern and western regions, both of which will reach the maximum in the first period in the future. There is a “negative crowding out effect” in the central region in the short term, but over time, from the perspective of the long-term dynamic relationship, the negative impact in the central region will gradually weaken. On the whole, R&D investment has a restraining effect on environmental regulation, but the contribution rate of the restraining effect is generally low. Unlike the previous literature [22, 25], this paper argues that R&D investment has a restraining effect on environmental regulation, that is, the increase in R&D investment will help reduce pollution costs to a certain extent, thus expanding the theoretical basis for achieving comprehensive sustainable development.

Finally, It is found that R&D investment has little influence on the “green degree” of technological innovation as a whole. The research in this paper shows thatthe contribution rate of R&D investment to the positive effect of green technological innovation is relatively weak, showing only a minor promotion effect in the eastern and western regions. The central region has not yet reflected the positive promotion effect. Green technology innovation has a strong reaction to R&D investment. There is a promotion effect in the eastern region that reaches the maximum in the first phase in the future, while there is a negative impact in the central and western regions. The negative impact in the central region is the strongest. In contrast to the conclusions of the current literature, the results of this study indicate that the phenomena of “central collapse” has always occurred and that the process of the central region’s ascent remains obstructed and protracted.

Based on the above research results, our conclusions have several important theoretical and practical contributions: (1) First, unlike previous literature, the research presented in this paper enhances our understanding of the relationship between environmental regulation, R&D investment, and the development of green technologies. And previous literature mainly explores the one-way influence relationship between the two, and rarely puts the three into the same framework for dynamic two-way research. (2) Second, we use the entropy method to establish a composite index of R&D investment and green technology innovation, which is important to contribute to the scientific accuracy of the results. (3) Finally, in a practical sense, this study refines different regions, adapts to the requirements of the development of different economic zones in China, and provides evidence support for the formulation of appropriate environmental regulations. Our findings are applicable to China and other developing countries or regions in the world, and can guide them to further improve environmental regulatory policies and green technology innovation and R&D decisions, so as to provide an effective way to achieve green and sustainable development.

Based on the above research conclusions, this paper puts forward the following relevant policy suggestions.

(1) The formulation of environmental regulation policies must be suitable for regional conditions. The government should avoid blindly improving the intensity of environmental regulation to ensure the green development of technological innovation in various regions according to local conditions. For the leading areas of R&D investment, environmental regulation should be strengthened appropriately. Meanwhile, it cannot ignore the “green” tendency of R&D investment. For the central region, regional resource endowments and comparative advantages should be combined. It should be considered that the government must guide enterprises to transform from seeking cost advantages to green technology innovation by means of “changing cages for birds”, introduce high-tech industries with a friendlier environment and higher technical level, promote industrial transformation and upgrading, and reduce the overall emission of local pollutants. These efforts will achieve a “win–win” outcome between high-quality economic development and high-level ecological environment protection.

(2) The bias against R&D investment of enterprises to green development should be addressed. Enterprises should comply with the requirements of green development. In terms of R&D investment, they should handle the relationship between product research and development and process optimization. They actively create the green brand effect of enterprises and are good at obtaining more benefits from pollution prevention and control. On the one hand, given the increasing seriousness of global environmental problems, the “backward effect” of environmental regulation is getting tighter and tighter. Enterprises have less room to maneuver in dealing with the “dilemma” between business activities and environmental protection. On the other hand, with the implementation of the comprehensive green transformation strategy of domestic economic and social development, the transformation of green products and the development of a pollution-free environment have increasingly become key elements of corporate publicity of social responsibility, brand improvement and continuous transformation and upgrading.

(3) Increasing the inclination of national innovation resources to the central and western regions is extremely urgent. Compared with the leading regions of technological innovation in the east, the central and western regions produce relatively more carbon emissions and pollutants in economic development. The “green degree” of regional overall technological innovation needs to be further improved. To promote the comprehensive green transformation of economic and social development and solve the problem of unbalanced regional green development, it is urgent to give certain policy privileges to local governments and enterprise innovation subjects in the central and western regions based on local characteristics to form a balanced development pattern of green innovation with “one place, one characteristic”. Simultaneously, the introduction of innovative talent to the central and western regions should be strengthened to meet the talent demand of characteristic industrial innovation and key green innovation fields in the central and western regions.

## Supporting information

S1 File (CSV)
